# 1‐Hydroxy‐xanthine derivatives inhibit the human Caf1 nuclease and Caf1‐containing nuclease complexes via Mg^2+^‐dependent binding

**DOI:** 10.1002/2211-5463.12605

**Published:** 2019-03-07

**Authors:** Blessing Airhihen, Lorenzo Pavanello, Gopal P. Jadhav, Peter M. Fischer, Gerlof Sebastiaan Winkler

**Affiliations:** ^1^ School of Pharmacy University of Nottingham UK; ^2^ School of Pharmacy Centre for Biomolecular Sciences University of Nottingham UK; ^3^ Present address: Department of Pharmacology School of Pharmacy Niger Delta University Wilberforce Island Nigeria; ^4^ Present address: Domainex Ltd Chesterford Research Park Little Chesterford, Saffron Walden, Essex UK; ^5^ Present address: School of Medicine Department of Clinical & Translational Sciences Creighton University Omaha NE USA

**Keywords:** 1‐hydroxy‐xanthine, Caf1/CNOT7 nuclease, Ccr4‐Not, deadenylase, Ribonuclease, thermal stability assay

## Abstract

In eukaryotic cells, cytoplasmic mRNA is characterised by a 3′ poly(A) tail. The shortening and removal of poly(A) tails (deadenylation) by the Ccr4‐Not nuclease complex leads to reduced translational efficiency and RNA degradation. Using recombinant human Caf1 (CNOT7) enzyme as a screening tool, we recently described the discovery and synthesis of a series of substituted 1‐hydroxy‐3,7‐dihydro‐1*H*‐purine‐2,6‐diones (1‐hydroxy‐xanthines) as inhibitors of the Caf1 catalytic subunit of the Ccr4‐Not complex. Here, we used a chemiluminescence‐based AMP detection assay to show that active 1‐hydroxy‐xanthines inhibit both isolated Caf1 enzyme and human Caf1‐containing complexes that also contain the second nuclease subunit Ccr4 (CNOT6L) to a similar extent, indicating that the active site of the Caf1 nuclease subunit does not undergo substantial conformational change when bound to other Ccr4‐Not subunits. Using differential scanning fluorimetry, we also show that binding of active 1‐hydroxy‐xanthines requires the presence of Mg^2+^ ions, which are present in the active site of Caf1.

**Abbreviations**
A.U.: arbitrary units
ADP: adenosine diphosphate
AMP: adenosine monophosphate
ATP: adenosine triphosphate
DMSO: dimethyl sulfoxide
DSF: differential scanning fluorimetry
FRET: Förster resonance energy transfer
IC_50_: half maximal inhibitory concentration
poly(A): polyadenosine
SDS/PAGE: sodium dodecyl sulphate‐polyacrylamide gel electrophoresis
SEM: standard error of the mean
*T*_m_: melting temperature

In eukaryotes, cytoplasmic mRNA is characterised by the presence of a 3′ poly(A) tail. Poly(A) tail length is broadly distributed with the median length varying from 27–28 nucleotides in yeast to 40–100 nucleotides in mammalian cells 1, 2. The poly(A) tail contributes to mRNA stability and translational efficiency, and the removal of the poly(A) tail is an important step in regulated mRNA degradation 3, 4. A key multi‐subunit enzyme complex involved in the controlled removal of the poly(A) tail is the Ccr4‐Not deadenylase complex that is composed of two catalytic nuclease subunits and six noncatalytic components. In mammalian cells, the large subunit CNOT1 is the scaffold of the complex 5, 6. Its N‐terminal domain interacts with CNOT11 and CNOT10, whose function is not well understood 6, 7. The MIF4G domain of CNOT1 is part of the nuclease module and binds to the Caf1 catalytic subunit (encoded by either *CNOT7* or *CNOT8*), which in turn interacts with the leucine‐rich repeat domain of the second catalytic subunit Ccr4 (encoded by *CNOT6* or *CNOT6L*) 8, 9. The DUF3819 domain of CNOT1 binds to CNOT9 10, 11, while the C‐terminal region of CNOT1 interacts with the CNOT2 and CNOT3 subunits forming the NOT‐module 12, 13. The noncatalytic subunits stimulate the activity of the nuclease module 14, 15 and form a platform for interactions with regulators of mRNA stability, including the GW182/TNRC6 component of the microRNA repression complex 16, 17, and the AU‐rich element binding protein tristetraprolin TTP (ZFP36) 18, 19, 20. Using genetically modified mice, specific physiological roles for a number of Ccr4‐Not components have been uncovered. The Caf1 nuclease subunit encoded by the *Cnot7* gene regulates bone formation by inhibiting osteoblast activity 21 and is also required for male fertility 22, 23. Deletion of one copy of *Cnot3*, a noncatalytic subunit of Ccr4‐Not, protects against obesity in mice 24. Mutations in Ccr4‐Not components have also been implicated in human disease. Nonsense mutations in *CNOT3* are associated with acute lymphoblastic leukaemia, whereas a recurrent missense mutation in *CNOT9* is found in metastatic melanoma 25, 26. Furthermore, mouse models of breast cancer have implicated Ccr4‐Not components in promoting metastatic cancer 27, 28. In case of Caf1, its enzymatic activity is required for this effect indicating that Caf1 inhibitors may be useful pharmacological tools to validate this protein as a possible drug target to prevent metastatic breast cancer 28.

Although much progress has been made in the past few years, the mechanism of poly(A) deadenylation by Caf1 and Ccr4 is still not fully understood. The Caf1 subunit, which contains an RNAse D/DEDD (Asp‐Glu‐Asp‐Asp) nuclease domain, appears dispensable in budding yeast, but makes a contribution to the activity of the complex in fission yeast and the fruit fly 29, 30, 31. On the other hand, the second catalytic nuclease subunit, the EEP (endonuclease–exonuclease–phosphatase) domain protein Ccr4, is the catalytic subunit in budding yeast and – like Caf1 – contributes to the activity in fission yeast and *Drosophila* 15, 32, 33, 34. The situation is more complex in human cells, where both Caf1 and Ccr4 appear to be required for activity of the nuclease module, which has much increased activity as compared to either the isolated Caf1 or Ccr4 subunit 35. Moreover, it has been proposed that the Caf1 and Ccr4 subunits have specialised roles, with Ccr4 required for the degradation of poly(A) bound by the poly(A)‐binding protein PABPC1, which inhibits the activity of Caf1 36, 37. On the other hand, it has been reported that the highly similar proteins BTG1 and BTG2 can mediate interactions between PABPC1 and Caf1, thereby promoting deadenylation by Caf1 38.

Recently, we reported the discovery and synthesis of a series of substituted 1‐hydroxy‐3,7‐dihydro‐1*H*‐purine‐2,6‐dione (1‐hydroxy‐xanthine) compounds as the first non‐nucleoside inhibitors of Caf1 39. Here, we report the further biochemical characterisation of these compounds by using two assays: (a) a chemiluminescence‐based assay for the detection of AMP, the product of the deadenylation reaction; and (b) differential scanning fluorimetry (thermal shift assay). Using these assays, we demonstrate that the 1‐hydroxy‐xanthine compounds inhibit the isolated human Caf1 enzyme with the same potency as human Caf1‐containing complexes that also contain the second catalytic subunit Ccr4. This suggests that the active site of Caf1 does not undergo substantial conformational changes when incorporated into the Ccr4‐Not complex. In addition, we show that active 1‐hydroxy‐xanthines require the presence of Mg^2+^ ions for binding, which provides experimental support for the proposed role of the *N*‐hydroxyimide moiety in coordinating Mg^2+^ ions present in the active site of Caf1. These experiments also demonstrate the value of the chemiluminescence‐based AMP detection assay and differential scanning fluorimetry as useful orthogonal assays that supplement our previously reported fluorescence‐based FRET assay 40 for the discovery and characterisation of inhibitors of the Caf1 nuclease enzyme.

## Materials and methods

### Reagents

1‐Hydroxy‐xanthine compounds **1**–**5** were described previously 39. The human Caf1/CNOT7 enzyme was expressed and purified from *Escherichia coli* BL21 (DE3) using procedures described before 40. The trimeric human nuclease module (BTG2‐Caf1/CNOT7‐Ccr4/CNOT6L) was obtained by co‐expression of the three proteins using plasmids pQE80L‐BTG2 and pACYC‐Duet1‐Strep‐CNOT6L‐CNOT7 and purified as described before 14, 35. The human pentameric central module (BTG2‐Caf1/CNOT7‐Ccr4/CNOT6L‐CNOT1‐CNOT9) of Ccr4‐Not was obtained by reconstitution of the complex after separate expression and purification of the nuclease module and the dimeric complex composed of CNOT1 (MIF4G and DUF3819 domains; amino acids 1093–1595) and CNOT9 (ARM domain; amino acids 33–283) using plasmid pACYC‐Duet1‐CNOT1‐CNOT9 as described before 14.

### Enzyme activity assays

Standard reaction conditions for deadenylase assays (10 μL) were as follows: 25 nm enzyme, 20 mm Tris/HCl pH 7.9, 50 mm NaCl, 2 mm MgCl_2_, 10% glycerol, 1 mm β‐mercaptoethanol and 1.0 μm 5′‐Flc‐labelled RNA substrate in nuclease‐free water as described before 40. Reactions containing inhibitory compounds also contained 5% DMSO. After incubation at 30 °C for 60 min, chemiluminescence‐based detection of AMP was carried out using the AMP‐Glo kit (Promega, Southampton, Hampshire, UK) 41, 42, 43. To this end, 5 μL of the enzyme reaction was mixed with an equal volume of AMP‐Glo reagent I in a white 96‐well half‐area multiwell plate (Corning Costar, reference 3693). After a further 60‐min incubation at room temperature, 10 μL AMP‐Glo reagent II was added. After another 60‐min incubation at room temperature, chemiluminescence was measured using a GloMax 96 multiwell plate luminometer (Promega). Data were exported to Microsoft Excel 2013 and analysed using graphpad prism (version 7).

### Differential scanning fluorimetry

Differential scanning fluorimetry (thermal stability assay) was carried out as described with minor modifications using an Agilent MX3005p instrument and the fluorescent dye SYPRO Orange (5000 × in DMSO, Thermo Fisher Scientific, Loughborough, Leicestershire, UK) 44. Standard assays (20 μL) contained the following: 2 μm purified Caf1/CNOT7 enzyme, 20 mm Tris/HCl pH 7.8, 50 mm NaCl, 10% glycerol, 1 mm β‐mercaptoethanol, 0.2 × SYPRO Orange dye, 100 μm compound, 5% DMSO. Assays carried out in the presence of divalent metal ions also contained 2 mm MgCl_2_.

Reactions were performed in thin‐wall, PCR grade 96‐well plates with V‐shaped wells. After sealing using optically clear lids, reactions were thoroughly mixed and collected at the bottom of the wells by centrifugation. Fluorescence scanning was carried out using a temperature gradient from 25 °C to 95 °C at 1 °C per minute using the FAM (492 nm) filter for excitation and the ROX (610 nm) filter for detection. Each experiment contained three technical replicates. Data were acquired using mxpro qpcr software (Agilent, Stockport, Cheshire, UK), exported to Microsoft Excel 2013, and analysed using publicly available macros for Microsoft Excel 2013 and templates for graphpad prism (version 7) as described 44.

### Statistical analysis

Standard errors of the mean were calculated using graphpad prism (version 7). Reported IC_50_ values were determined based on at least three independent experiments each containing at least two technical replicates. Reported Δ*T*_m_ values were calculated based on at least three independent experiments, each containing three technical replicates. *P* values were calculated using a two‐tailed *t*‐test (graphpad prism, version 7).

## Results and Discussion

### Chemiluminescence‐based detection of AMP as a sensitive assay for deadenylase activity

**Figure 1:**
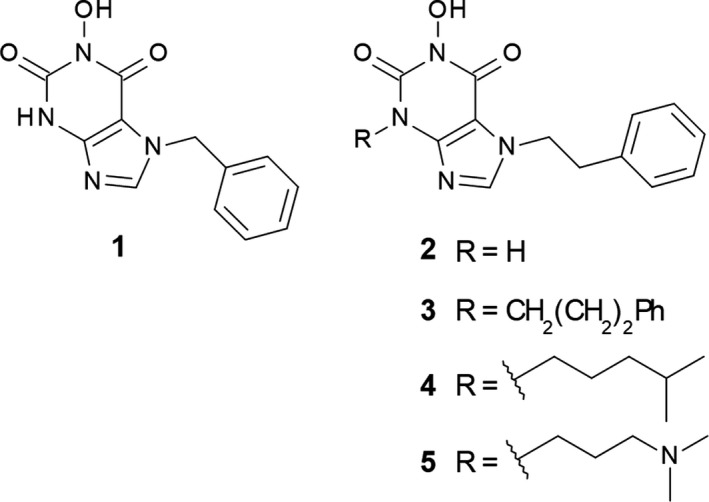
Structure of 1‐hydroxy‐xanthine derivatives **1–5.**

**Figure 2:**
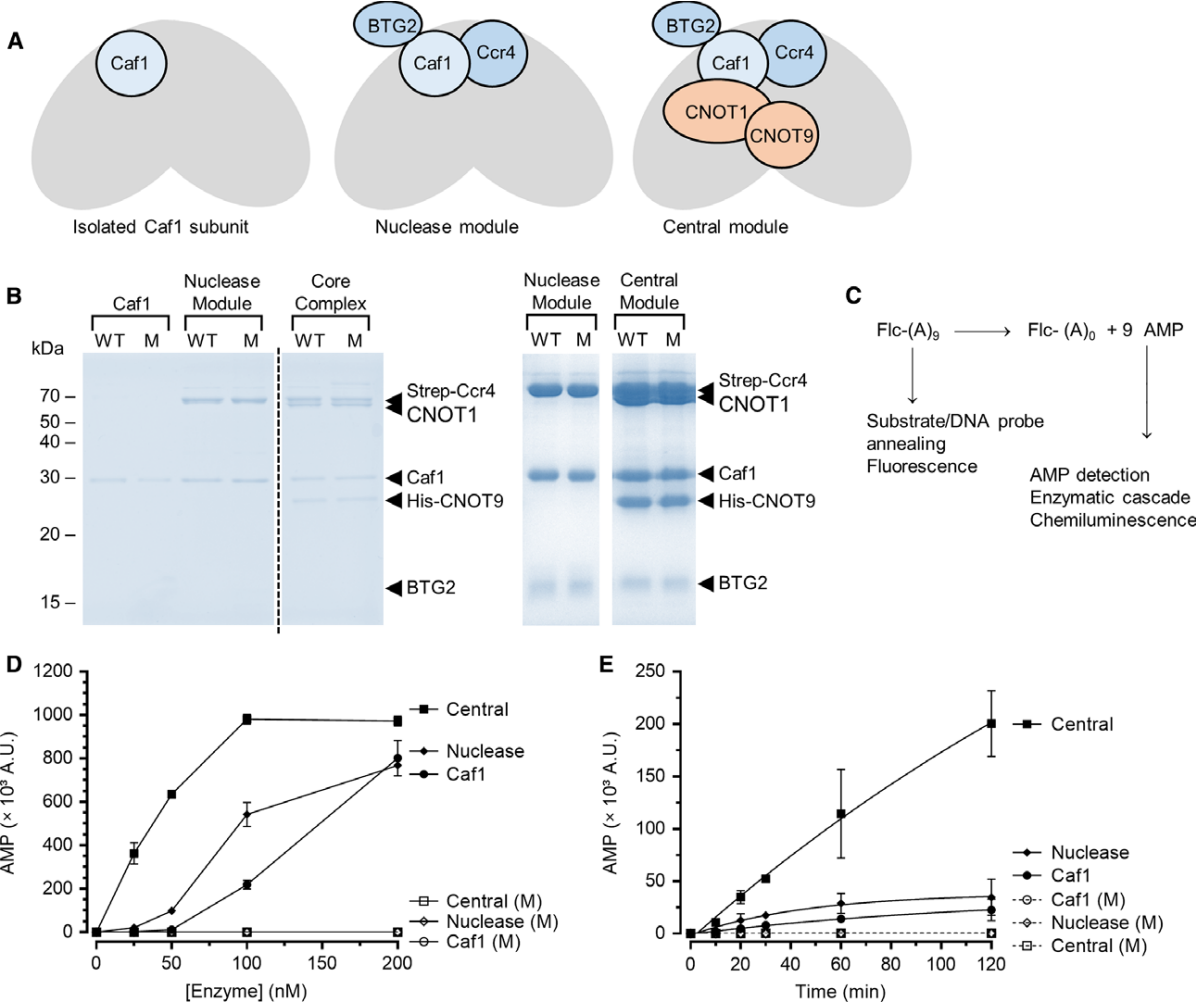
Application of a chemiluminescence‐based AMP detection system as a quantitative assay for deadenylation activity. (A) Schematic overview of Caf1 and Caf1‐containing complexes. (B) Purified recombinant Caf1 and Caf1‐containing complexes. WT, wild‐type; M, mutant protein preparations containing the D40A amino acid substitution in Caf1 or the D40A substitution in Caf1 and the E240A substitution in Ccr4 (nuclease and central modules). *Left panel*, purified proteins (5 μg) were separated by 14% SDS/PAGE and stained with Coomassie Brilliant Blue. *Right panel,* purified proteins (20 μg) were separated by 14% SDS/PAGE and stained with Coomassie Brilliant Blue. (C) Schematic of the deadenylation reaction and the use of activity assays based on RNA substrate (*left*) and AMP product (*right*) detection. The fluorescence‐based assay was described before 40. (D) Activity of Caf1 and Caf1‐containing complexes using chemiluminescence‐based AMP detection. Protein samples were incubated with a synthetic RNA substrate at 30 °C for 60 min before the detection of AMP levels. (E) Time‐course analysis of Caf1 and Caf1‐containing complexes. Protein samples (25 nm) were incubated with a synthetic RNA substrate containing 9 terminal adenosine residues (1.0 μm) at 30 °C. A.U., arbitrary units. Error bars represent the standard error of the mean (*n *= 3).

To determine the activity of 1‐hydroxy‐xanthine compounds (Fig. 1) versus human Caf1 and Caf1‐containing complexes that also contain the second catalytic subunit Ccr4, we first purified the isolated Caf1 protein, which displays relatively weak deadenylase activity, the trimeric BTG2‐Caf1‐Ccr4 nuclease module, which displays relatively robust nuclease activity, and the pentameric central module, which displays relatively high enzymatic activity (Fig.**** 2A). The proteins were expressed in *Escherichia coli* and were purified using affinity chromatography as described before (Fig.**** 2B) 14, 35, 40. We then evaluated the use of chemiluminescence‐based detection of AMP 41, 42, 43 as an activity assay of Caf1 and Caf1‐containing complexes. We chose this assay as a potential assay that is complementary to the fluorescence‐based assay described before (Fig.**** 2C) 40. Importantly, the AMP detection assay is suitable for multiwell plates, allowing the analysis of many compounds in parallel, and does not depend on time‐consuming analysis of reaction products by gel electrophoresis.

We used a synthetic 15‐mer RNA oligonucleotide with nine adenosines at the 3′ end as the enzyme substrate 40. After incubation with varying concentrations of the Caf1 enzyme, AMP was detected in a two‐step procedure. In the first step, AMP was enzymatically converted to ADP. Then, enzymatic conversion of ADP to ATP in the presence of luciferase and luciferin resulted in chemiluminescence proportional to the amount of AMP produced in the reaction. Robust levels of AMP were detected at enzyme concentrations below 100 nm, which is substantially lower as compared to the amount of enzyme required for the fluorescence‐based assay (Fig.**** 2D,E) 40. Only very low levels of AMP were produced in the presence of the catalytically inactive variant D40A, and a signal/background ratio > 10 was achieved at wild‐type enzyme concentrations > 25 nm (Fig.**** 2D,E). When the activity of Caf1‐containing complexes was determined, much increased levels of AMP were detected in agreement with our previous findings that the activity of the nuclease and central modules of Ccr4‐Not display higher activity than the isolated Caf1 subunit 14, 35. The activity determined in the presence of Caf1‐containing complexes was specific, because only very low levels of AMP were detected in the presence of Caf1‐containing complexes with inactive Caf1 (D40A) and Ccr4 (E240A) catalytic subunits.

Together, these experiments demonstrate that chemiluminescence‐based detection of AMP is a highly sensitive assay that is suitable for the evaluation of the enzymatic activity of Caf1 and Caf1‐containing complexes.

### 1‐Hydroxy‐xanthine compounds inhibit Caf1 and Caf1‐containing complexes with similar potency

**Figure 3:**
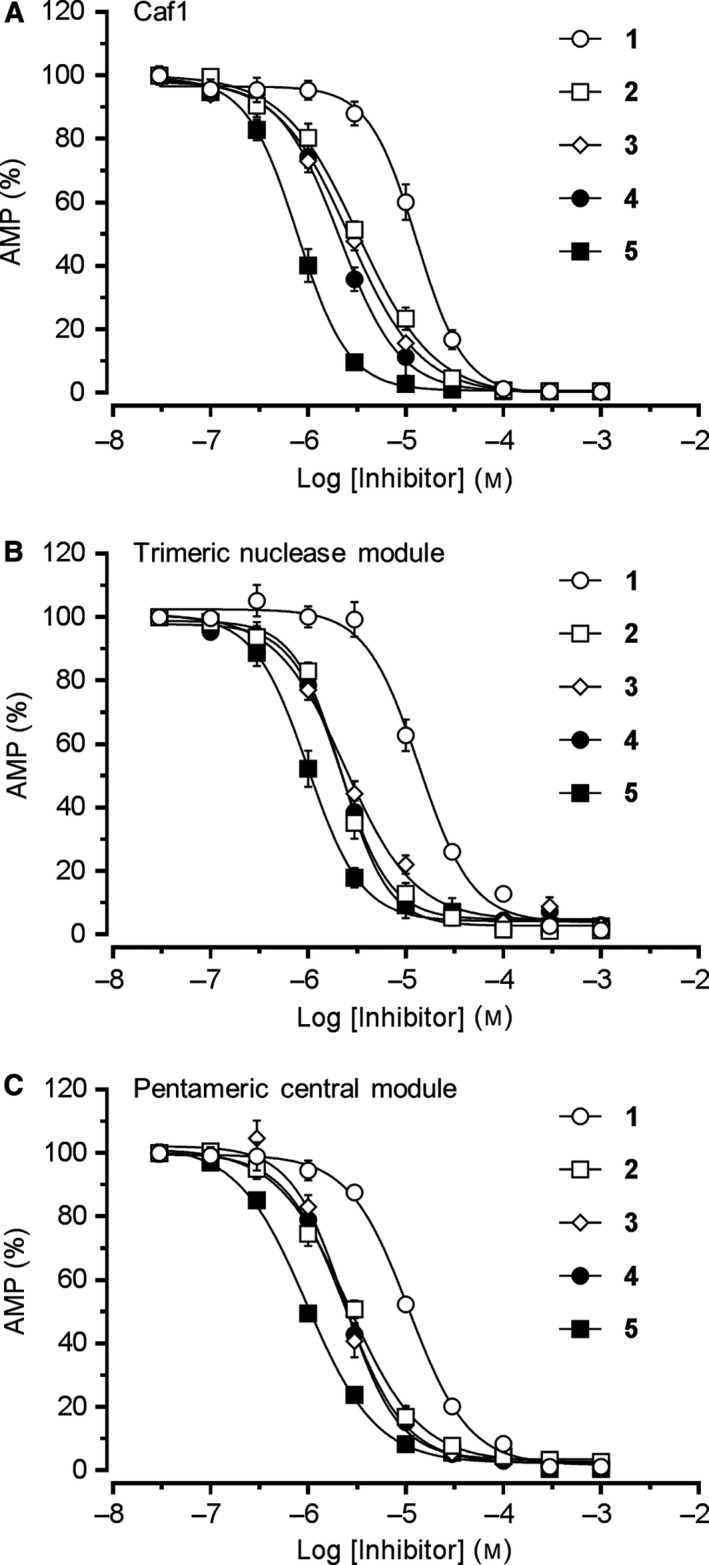
Inhibition of Caf1 and Caf1‐containing complexes by 1‐hydroxy‐xanthines. (A) Inhibition of the isolated Caf1 subunit. (B) Inhibition of a trimeric BTG2‐Caf1‐Ccr4 nuclease module of Ccr4‐Not. (C) Inhibition of a pentameric BTG2‐Caf1‐Ccr4‐CNOT1‐CNOT9 central module of Ccr4‐Not. Enzyme preparations (25 nm) were incubated with a synthetic RNA substrate containing nine terminal adenosine residues (1.0 μm) at 30 °C for 60 min. Error bars represent the standard error of the mean (*n* = 3).

To test whether the assay can be used as a quantitative assay for the characterisation of small‐molecule inhibitors, we next determined the IC_50_ value of five 1‐hydroxy‐xanthine compounds **1**–**5**, which were previously characterised using the fluorescence‐based assay and inhibit Caf1 with IC_50_ values ranging from 0.59 ± 0.11 μm to 10.4 ± 0.4 μm (Fig.**** 1 and Table 1) 39. Importantly, these compounds do not display activity towards the catalytic domain of Ccr4 39. To determine the IC_50_ values, we used conditions where the reaction product increased proportionally with time and substrate concentrations were not limiting (Fig. 2E, *t* = 60 min). As shown in Fig.**** 3A and Table 1, the IC_50_ values determined using chemiluminescence‐based AMP detection closely matched the values previously obtained using the fluorescence‐based assay. Based on these results, we conclude that both assays are suitable for the characterisation of the activity of compounds that inhibit deadenylase enzymes and result in comparable IC_50_ values.

**Table 1:** Activity of 3,7‐disubstituted‐1‐hydroxy‐1,7‐dihydro‐1*H*‐purine‐2,6‐diones

Cmpd	IC_50_ (μm) *vs* Caf1 (Reference)^a^	IC_50_ (μm) *vs* Caf1^b^	IC_50_ (μm) *vs* trimeric Caf1‐complex^c^	IC_50_ (μm) *vs* pentameric Caf1‐complex^d^	Differential scanning fluorimetry	
					Δ*T*_m_ (°C) Caf1 (no MgCl_2_)^e^	Δ*T*_m_ (°C) Caf1 (MgCl_2_)^f^
**1**	10.4 ± 0.4	12.7 ± 0.9	13.6 ± 0.6	10.8 ± 0.2	−0.4 ± 0.3	2.4 ± 0.3
**2**	1.5 ± 0.3	3.2 ± 0.4	2.3 ± 0.2	2.6 ± 0.3	0.6 ± 0.4	4.1 ± 0.3
**3**	2.1 ± 0.3	2.7 ± 0.3	2.4 ± 0.2	2.4 ± 0.1	0.5 ± 0.5	3.3 ± 0.2
**4**	1.7 ± 0.4	2.2 ± 0.3	2.2 ± 0.1	2.5 ± 0.4	0.6 ± 0.6	3.4 ± 0.4
**5**	0.59 ± 0.11	0.79 ± 0.09	1.0 ± 0.2	0.98 ± 0.07	−0.2 ± 0.3	4.7 ± 0.3

a IC_50_ values determined using a fluorescence‐based assay were taken from Jadhav *et al*. 39.

b IC_50_ values were determined using chemiluminescence‐based AMP detection in the presence of monomeric Caf1/CNOT7 enzyme.

c IC_50_ values were determined using chemiluminescence‐based AMP detection in the presence of trimeric BTG2‐Caf1‐Ccr4 nuclease module.

d IC_50_ values were determined using chemiluminescence‐based AMP detection in the presence of pentameric BTG2‐Caf1‐Ccr4‐CNOT1‐CNOT9 central module.

e Differential scanning fluorimetry. Δ*T*_m_ values were determined by differential scanning fluorimetry in the presence of Caf1/CNOT7 and the indicated compound.

f Differential scanning fluorimetry. Δ*T*_m_ values were determined by differential scanning fluorimetry in the presence of Caf1/CNOT7, 2 mm MgCl_2_ and the indicated compound. Indicated are the mean ± standard error of the mean (*n* = 3).

To determine whether our recently developed series of 1‐hydroxy‐xanthines inhibit Caf1‐containing complexes with similar potency compared to the isolated Caf1 subunit, we next determined the activity of compounds **1**–**5** versus the trimeric nuclease module and the pentameric central module of the Ccr4‐Not complex (Fig.**** 3B,C). As shown in Fig.**** 3 and Table 1, the selected 1‐hydroxy‐xanthines displayed similar activity versus the trimeric nuclease module (Fig.**** 3B) as well as the pentameric central module (Fig. 3C). These results indicate that the active site of the isolated Caf1 subunit does not undergo substantial conformational changes upon association with other Ccr4‐Not subunits, including the Ccr4 ribonuclease subunit, whose leucine‐rich repeat (LRR) domain interacts directly with the Caf1/CNOT7 subunit, or the CNOT1 subunit, which binds to Caf1 via the MIF4G domain. Moreover, the compounds fully inhibited the activity of Caf1 and the Caf1‐containing nuclease complexes showing that the Caf1 subunit is indispensable for deadenylase activity in the context of the nuclease and central modules, even in the presence of the second catalytic nuclease subunit Ccr4.

### 1‐Hydroxy‐xanthine compounds require the presence of Mg^2+^ ions for binding

**Figure 4:**
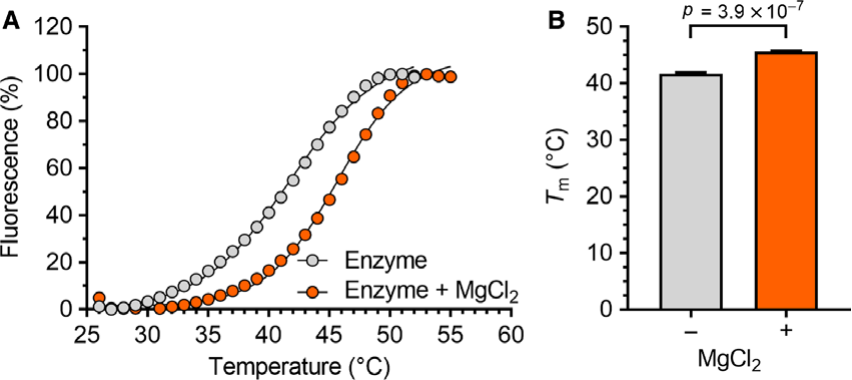
Increased thermal stability of Caf1 in the presence of Mg^2+^ ions. (A) Differential scanning fluorimetry of Caf1 in the absence and presence of MgCl_2_. Caf1 (2 μm) was incubated in the absence or presence of 2 mm MgCl_2_ and the SYPRO Orange dye. Fluorescence scanning was carried out using a temperature gradient from 25 °C to 95 °C. The melting temperature *T*_m_ was determined as described 44. (B) Thermal stability of Caf1 in the absence and presence of MgCl_2_. Δ*T*_m_ = 3.9 ± 0.2 °C; mean ± SEM (*n *= 7). Error bars indicate the standard error of the mean (n = 7).

**Figure 5:**
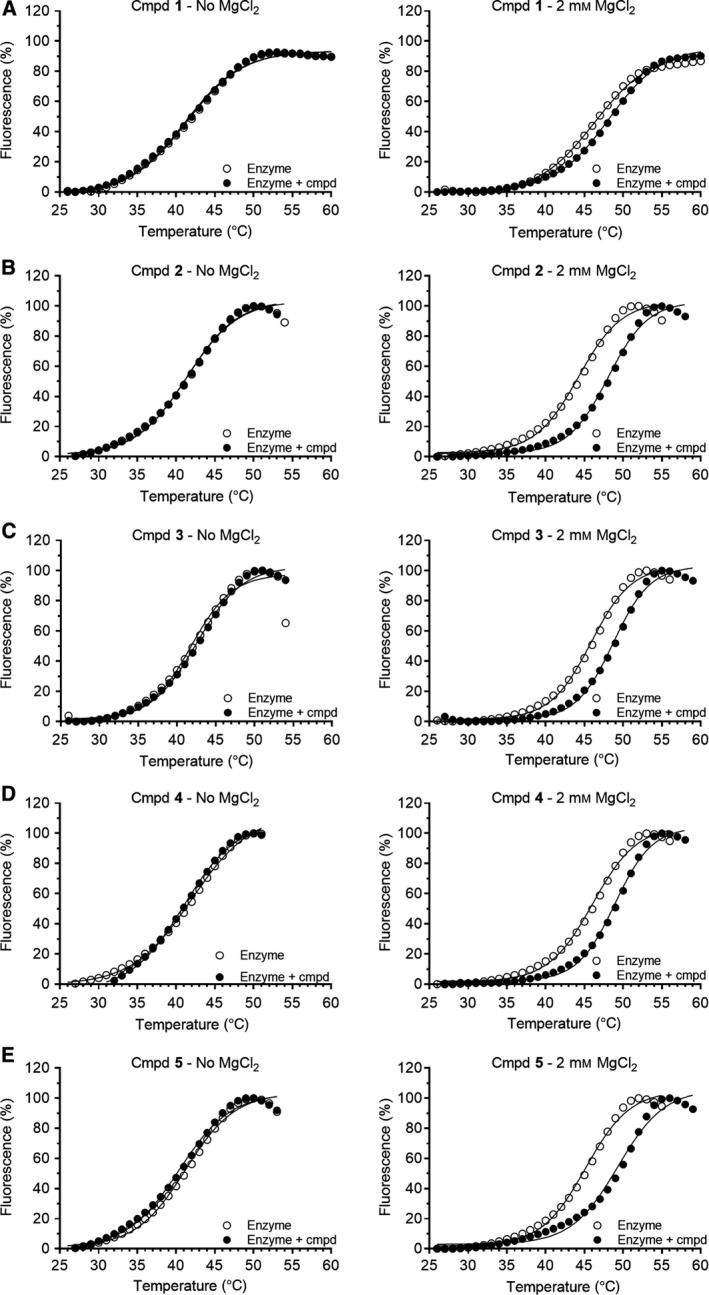
Binding of 1‐hydroxy‐xanthines requires the presence of Mg^2+^ ions. (A‐E) Representative experiments showing binding of compounds **1**–**5** to Caf1 in the absence of Mg^2+^ (*left panels*) and 2 mm MgCl_2_ (*right panels*). Compounds (100 μm) were incubated with Caf1 (2 μm) and the SYPRO Orange dye. Fluorescence scanning was carried out using a temperature gradient from 25 °C to 95 °C. The melting temperature *T*_m_ was determined as described 44.

Having established that 1‐hydroxy‐xanthines inhibit Caf1 and Caf1‐containing complexes with similar potency, we next aimed to obtain further information about their mode of binding. Specifically, we aimed to establish if binding of 1‐hydroxy‐xanthines requires the presence of Mg^2+^ ions in the active site as suggested by our proposed binding mode 39 or that of related compounds containing an *N*‐hydroxyimide moiety to the influenza endonuclease PA protein and the human DNA structure‐specific endonuclease FEN‐1 45, 46, 47, 48. To this end, we evaluated the use of differential scanning fluorimetry (DSF), which is based on increased thermal stability of a protein induced upon binding of small‐molecule ligands 44, 49, 50. First, we determined the melting temperature *T*_m_ of Caf1 in the absence and presence of MgCl_2_ and observed a robust increase in the *T*_m_ of Caf1 in the presence of Mg^2+^ ions (Fig.  4; Δ*T*_m_ = 3.9 ± 0.2 °C; mean ± SEM, *n *= 7). Encouraged by these results, we then carried out DSF using Caf1 in the presence of 1‐hydroxy‐xanthines **1**–**5**. In the absence of MgCl_2_, no significant changes in the melting temperatures were observed in the presence of compounds **1**–**5** as compared to enzyme only (*P* > 0.05; Table 1 and Fig. 5). By contrast, significant changes in the thermal shifts were observed in the presence of compound **1**–**5** and MgCl_2_ as compared to control reactions containing enzyme and MgCl_2_ only (P < 0.05). These observations suggest that binding of 1‐hydroxy‐xanthines requires the presence of Mg^2+^ ions, which are present in the active site of Caf1.

### Correlation between thermal shifts and IC_50_ values

**Figure 6:**
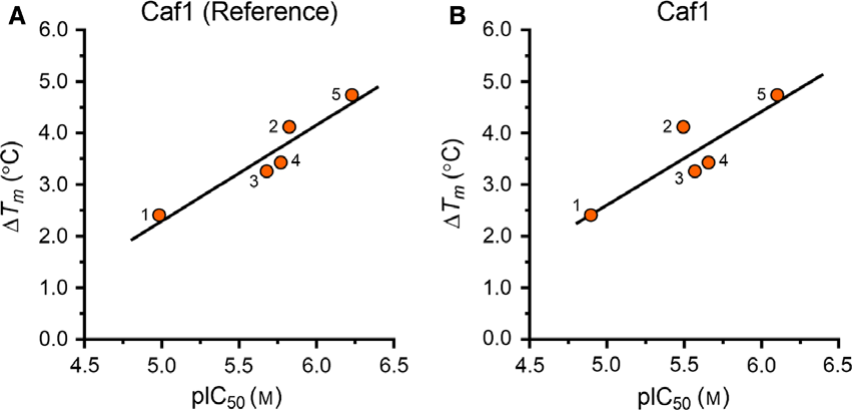
Correlation between thermal shifts (Δ*T*_m_) observed in the presence of MgCl_2_ and inhibitory activity of compounds **1**–**5**. (A) Correlation between thermal shift and pIC_50_ values determined using the isolated Caf1 protein and the fluorescence‐based assay (*r* ^2^ = 0.90). (B) Correlation between thermal shift and pIC_50_ values determined using the isolated Caf1 protein and the chemiluminescence‐based AMP detection assay (*r* ^2^ = 0.78). Linear regression was carried out using graphpad prism (version 7.0).

Because it has been shown that thermal shift assays can provide quantitative information about the binding affinity of enzyme ligands 50, we next considered whether the observed thermal shifts correlated with the IC_50_ inhibitory concentrations of compounds **1**–**5**. To this end, we plotted the Δ*T*_m_ values observed in the presence of MgCl_2_ against the corresponding IC_50_ values. As shown in Fig. 6, we found a high correlation between the Δ*T*_m_ values and the pIC_50_ values obtained using the isolated Caf1 enzyme (*r* ^2^ = 0.90 using the fluorescence‐based FRET assay and *r* ^2^ = 0.78 using the chemiluminescence‐based AMP detection assay). This indicates that the observed thermal shifts provide quantitative information about the relative potency of compounds **1**–**5** (Fig.**** 6) versus the Caf1 nuclease. Thus, we show that differential scanning fluorimetry is not only useful to provide information about the binding mode of Caf1 inhibitors, but may also be used as an additional screening tool to characterise and identify new compounds that inhibit the Caf1 nuclease.

In summary, the results reported here provide insight into the mechanism of inhibition of the Caf1 deadenylase enzyme by 1‐hydroxy‐xanthines. First, we show that compounds **1**–**5** inhibit the isolated enzyme with similar potency as Caf1‐containing complexes, which also include the Ccr4 nuclease subunit. It is notable that the activity of the Caf1‐containing complexes was completely inhibited at higher compound concentrations, even though the compounds do not affect Ccr4 39. This indicates that Caf1 is essential for the activity of complexes containing both Caf1 and Ccr4 nucleases. The observation that the nuclease complexes can be inhibited by small molecules extends our previously reported findings, which showed that the activity of the human BTG2‐Caf1‐Ccr4 nuclease complex requires the activity of both Caf1 and Ccr4, and that inactivating amino acid substitutions in either Caf1 or Ccr4 are sufficient to abolish the activity of the complex 35. This contrasts with the finding that Caf1 activity is not essential for the activity of complexes containing the Caf1 and Ccr4 nucleases from the fission yeast *S. pombe* and the fruit fly *Drosophila* and residual activity is observed upon inactivation of Caf1 in those cases 15, 33. Secondly, we showed that binding of compounds **1**–**5** requires the presence of Mg^2+^ ions, which bind in the active site of Caf1. This is in agreement with the proposed role of the *N*‐hydroxyimide moiety in 1‐hydroxy‐xanthines and related compounds forming ion–dipole interactions with the Mg^2+^ ions 45, 46, 47, 48.

In addition to these findings, two further assays for the evaluation of Caf1 inhibitors are presented here. The chemiluminescence‐based detection of AMP provides an orthogonal assay for our previously reported fluorescence‐based assay that does not depend on gel‐based product analysis. While the AMP detection method has some disadvantages, including the relative instability of the signal and the dependence on an enzymatic cascade for detection of AMP, which requires additional time‐consuming incubation steps, it benefits from increased sensitivity, a greater dynamic range and increased signal‐to‐noise ratios. In addition, we show that the widely used thermal shift assay is suitable for the analysis of inhibitor binding to Caf1 and also provides information about the binding mode of the inhibitor–enzyme complexes.

## Author contributions

GSW conceived the experiments. BA, LP and GSW designed the experiments. BA and LP carried out the experiments. GPJ and PMF contributed unique reagents. BA, LP and GSW analysed the data. BA, LP, PMF and GSW contributed to the preparation of the manuscript.

## Conflict of interest

The authors declare no conflict of interest.
